# Therapeutic efficacy of JNJ-49214698, an RSV fusion inhibitor, in RSV-infected neonatal lambs

**DOI:** 10.1099/jgv.0.002056

**Published:** 2024-12-11

**Authors:** Sarhad Alnajjar, Alejandro Larios-Mora, Albert Van-Geelen, Jack Gallup, Anil Koul, Peter Rigaux, Dirk Roymans, Mark Ackermann

**Affiliations:** 1 Department of Comparative Biomedical Sciences, School of Veterinary Medicine, Faculty of Health and Medical Sciences, University of Surrey, Guildford GU2 7AL, UK; 2 Lambcure, LLC, Portland, OR, USA; 3 Department of Veterinary Pathology, College of Veterinary Medicine, Iowa State University, Ames, IA, USA; 4 National Animal Disease Center, Ames, IA, USA; 5 Vaxxinova, Worthington, MN, USA; 6 Respiratory Infections Discovery, Janssen Infectious Diseases, Beerse, Belgium

**Keywords:** acute lower respiratory tract infection, antiviral, fusion inhibitor, neonatal lamb model, respiratory syncytial virus, RSV, small-molecule fusion inhibitor

## Abstract

Respiratory syncytial virus (RSV) is a leading cause of respiratory infection, hospitalization and death in infants worldwide. No fully effective RSV therapy using direct antivirals is marketed. Since clinical efficacy data from naturally infected patients for such antivirals are not available yet, animal studies are indispensable to predict therapeutic intervention. Here, we report the impact of an RSV fusion inhibitor, JNJ-49214698, on severe RSV-associated acute lower respiratory tract infection (ALRTI) in neonatal lambs. Randomized animals were treated once daily with 25 mg/kg JNJ-49214698, starting either before RSV infection, 1 day post-infection or as late as peak lung viral load on Day 3 post-infection. Treatment efficacy was assessed by scoring clinical signs of illness, development of RSV-induced gross and microscopic lung lesions and measuring virus titres in the lungs. Treatment with JNJ-49214698 was very effective in all treatment groups. Even in animals for which treatment was delayed until peak viral load was reached, a reduced amount and severity of gross and microscopic lesions, as well as RSV titres and RNA levels, were found. These results strongly suggest that treatment with small-molecule fusion inhibitors is an effective strategy to treat patients who are diagnosed with an RSV-induced ALRTI.

**Impact Statement**

JNJ-49214698, a recently discovered small-molecule RSV fusion protein inhibitor, effectively diminishes RSV infection and reduces RSV-associated acute lower respiratory tract infection. JNJ-49214698 inhibits multicyclic RSV infection and reduces RSV titre, RNA and disease severity. Thus, treatment was effective even in established RSV acute lower respiratory tract infections, providing evidence for the broad window of opportunity to treat RSV infection.

## Introduction

Respiratory syncytial virus (RSV) is one of the leading causes of acute lower respiratory tract infection (ALRTI). Globally, it is estimated that 33.8 million cases of ALRTI associated with RSV infection occur yearly in children under the age of five. Globally, RSV-associated ALRTI is a major cause of hospital admissions, with an estimated 3.4 million cases annually, and RSV-associated mortality is estimated to be 66 000–199 000 in children under the age of five, mainly occurring in developing countries [1]. Furthermore, post-bronchiolitis development of wheezing syndrome is sometimes observed, and although still under debate, it was suggested that the development of asthma could be associated with RSV infection [2,3]. RSV is also one of the leading causes of respiratory infection in the elderly and high-risk adults [4,5]. A recent study showed that 3% of pneumonia cases in human adults are caused by RSV [6], and with the widespread use of influenza vaccination, RSV has a similar disease burden in the elderly [4].

Despite decades of investigation, an RSV vaccine has recently been approved in the US, with some limitations in other countries [7,8]. Seasonal prophylaxis, i.e. administering treatment before the RSV season with the monoclonal antibody palivizumab and the recently approved nirsevimab, is restricted to high-risk infants in developed countries [9,10]. The disease-preventive effects of palivizumab (Synagis), which targets the RSV fusion protein, in these patients were demonstrated by a significant reduction in hospitalization rate, hospital residence time, admission to the intensive care unit and days requiring oxygen therapy [11]. However, the therapeutic efficacy of antibodies like palivizumab in infants hospitalized with an established RSV-associated ALRTI is questionable, i.e. failure to reduce viral load and disease severity in RSV hospitalized patients [12,14]. Other non-antiviral drugs, like bronchodilators, corticosteroids and antibiotics, often used but not indicated to treat RSV-associated bronchiolitis, do not seem to confer sufficient therapeutic benefit either, leaving supportive care essentially as the primary treatment option [15,16].

New treatments are needed to decrease the medical consequences related to RSV-associated ALRTI. Data on the impact of small-molecule direct antivirals on RSV disease manifestation in naturally infected hospitalized patients or outpatients are not available, and as such, reliable modelling of human RSV disease remains a necessary step in the search for novel therapies.

The development of an RSV experimental human infection model has provided a new tool to obtain human proof-of-concept efficacy data of RSV direct antivirals early in the clinical development pipeline [17,19]. However, volunteers are often young, healthy adults, and infection is studied only in the upper respiratory tract, limiting the information about the efficacy of therapeutics in treating ALRTI. Therefore, the recent development of a fully replicative neonatal lamb model for RSV has presented a significant step forward in generating reliable preclinical efficacy data and improving the selection of the proper clinical candidate molecules [20]. Neonatal lambs are susceptible to human as well as ovine and bovine strains. Similarities between the pulmonary and immunological systems of lambs and humans make neonatal lambs an excellent model for studying human RSV disease and the impact of new potential RSV therapeutics [21,22]. Moreover, neonatal lambs infected with RSV develop clinical symptoms including, but not limited to, fever, tachypnoea or increased expiratory effort, wheezing and lethargy, and develop mild to moderate bronchiolitis and pneumonia. Neonatal lambs' innate and adaptive immune responses are very similar to human infants. As in human infants, RSV infection in lambs induces variable severity and clinical symptoms. Although not common, some lambs have severe ALRTI that may lead to death if not treated [23,24].

A promising approach to inhibit RSV is targeting the viral fusion (F) protein [25,26]. Targeting viral fusion protein is an effective strategy in many approved antiviral treatments [27]. A few small-molecule RSV fusion inhibitors are currently being evaluated in early-stage clinical trials, but their clinical impact on RSV-associated ALRTI in hospitalized infants is yet unknown [28,31]. Therefore, this study aims to evaluate the therapeutic impact of an experimental RSV fusion inhibitor, JNJ-49214698, on RSV-associated ALRTI using a lamb model of RSV infection. This is a supplementary study of the previously published data [26].

## Methods

See online Supplementary Data for detailed methods.

### Compound and dosing

JNJ-49214698 was discovered and synthesized by Janssen Infectious Diseases (Beerse, Belgium). The compound was formulated in 10% acidified hydroxypropyl-β-cyclodextrin [10% HP-β-CD+HCl, pH 2 (vehicle)] at 6.25 mg/ml prior to dosing and stored throughout the study at 4 °C. The compound was dosed orally by catheter-mediated orogastric gavage at 4 ml/kg body weight (25 mg/kg) once daily. Dose selection in this study was aimed to reach the highest possible safe exposure in the animals to maximize the likelihood of obtaining efficacy while avoiding toxic side effects. A daily oral dose of 25 mg/kg was selected based on the antiviral activity of JNJ-49214698 [EC_50_=0.4 ng/ml (0.8 nM) and EC_90_=2.4 ng/ml (4.8 nM)], JNJ-49214698 exposure levels obtained in neonatal lambs at different doses during a separate pharmacokinetic (PK) study and an observed lack of toxicity of JNJ-49214698 at least until C_max_=17 167 ng/ml and AUC_0-24h_ = 139 993 ng h/ml in a 5 day repeated dose rat tolerance study (unpublished data).

### Animals

Total 21 colostrum-deprived neonatal lambs (Suffolk, Polypay, Dorsett cross) aged 1–3 days and 2–7 kg body weights were obtained for this experiment. Animal use was approved by the Institutional Animal Care and Use Committee of Iowa State University. RSV-infected lambs were kept in a separate room from the non-infected animals in the Livestock Infectious Disease Isolation Facility. Lambs were fed an iodide-free lamb milk replacer diet (Milk Products Inc., Chilton, WI, USA) [29] and were treated with Naxcel (Ceftiofur sodium, Pfizer) intramuscular once daily to reduce/prevent secondary bacterial infections.

### Experimental design

Lambs were randomly assigned to five different groups. Three groups (Px, Tx-1 and Tx-2) were infected with RSV and treated with JNJ-49214698. The first group (Px, *n*=4) was treated prophylactically 1 day before the RSV challenge and then daily afterwards until Day 5 post-infection (p.i.). The second (Tx-1, *n*=5) and the third (Tx-2, *n*=5) groups were treated 1 day and 3 days after viral challenge and daily afterwards up until Day 5 p.i., respectively. The vehicle group (*n*=4), serving as a positive RSV control group, was infected with RSV but received treatment with vehicle only. The No RSV group (*n*=3) served as the negative RSV control group and was aerosolized with RSV-free, HEp-2 cell-conditioned media. It also received a vehicle. All lambs were euthanized at Day 6 p.i., and all endpoints were measured after euthanasia.

### RSV infection

Lambs were infected with RSV strain M37, purchased from Meridian BioSciences (Memphis, TN, USA). This strain is a wild-type A RSV isolated from the respiratory secretions of an infant hospitalized for bronchiolitis [32,33]. M37 was grown in HEp-2 cells in our laboratory and stored at −80 °C in media containing 20% sucrose [34]. Total 6 ml of 1.27×10^7^ infectious focus forming unit (IFFU) /ml in media containing 20% sucrose or cell-conditioned mock media (also containing 20% sucrose) was nebulized using PARI LC Sprint™ nebulizers to each lamb over 25–30 min, resulting in the total inhalation of about 3 ml by each lamb [35].

### Assessed parameters

JNJ-49214698 exposure in plasma, broncho alveolar lavage fluid (BALF) and lung homogenate samples were assessed using a method based on protein precipitation and HPLC/MS/MS analysis. Lambs were monitored for the appearance of any signs of illness and respiratory-associated clinical signs (respiratory rate, wheezing, expiratory effort) as done previously [36]. Because clinical signs are variable in lambs and challenging to score in terms of severity, an accumulative clinical score to summarize the overall distress per group was applied by adding up the number of the scored clinical signs observed in each of the individual lambs. Quantitative reverse transcription polymerase chain reaction (qRT-PCR) was done to measure the RSV mRNA expression in BALF and lung homogenate samples as previously done in our lab [34,36, 37]. IFFU assay was used to determine the viable RSV in BALF samples as previously described [36]. For the pathological study, the per cent of lung parenchyma involved with RSV-induced lesions was measured to evaluate the RSV gross lesion. Histologically, haematoxylin-eosin-stained sections were examined via light microscope as described previously [36] with some modification. Lung lesions were scored according to an integer-based score of 0–4 for each parameter (bronchiolitis, syncytial cells, epithelial necrosis, epithelial hyperplasia, peribronchial lymphocytic infiltration, perivascular lymphocytic infiltration, neutrophils), with 4 as the highest score. Then, a final score (accumulative histo lesion score) was assigned by adding up the scores from the seven individual parameters, resulting in final accumulative scores ranging from 0 to 28, representing the total RSV-associated lesion in each tissue section. For further evaluation of the JNJ-49214698 therapeutic efficacy, immunohistochemical staining was performed to evaluate RSV antigen (Fusion protein) expression in the lung tissue section as previously described in our laboratory [36,38, 39]. Formalin-fixed paraffin-embedded tissue sections were used for the RNAscope detection of RSV mRNA *in situ*. A probe designed to the hRSV M37 nucleoprotein gene (accession number KM360090) was used (Probe-V-RSV-NP, Advance Cell Diagnostic, Catalogue number 439866). The assay was performed according to the manufacturer’s manual [user manual document number 320497; RNAscope® 2.0 HD Detection Kit (BROWN) User Manual PART 2]. Sections were examined under a light microscope, and the number of bronchioles and alveoli containing the positive signal was counted. The number of positive bronchioles and alveoli per tissue section was then assigned a score according to the simple integer-based scale of 0=no positive alveoli/bronchioles, 1=1–10 positive alveoli/bronchioles, 2=11–39 positive alveoli/bronchioles, 3=40–99 positive alveoli/bronchioles, 4=>100 positive alveoli/bronchioles.

### Statistical analysis

Statistical analysis was completed by using the Kruskall–Wallis test for non-parametric parameters such as accumulative microscopic lesion scoring, immunohistochemistry and RNAscope integer-based scores, followed by Dunn’s post-hoc test for multiple comparisons. One-way ANOVA followed by Dunnett multiple comparisons test was used to compare the treated groups to the RSV-infected non-treated control group for gross lesion scores and viral titre analyses by qRT-PCR.

## Results

### JNJ-49214698 efficiently distributes to different lung compartments of neonatal lambs

**Fig. 1.**
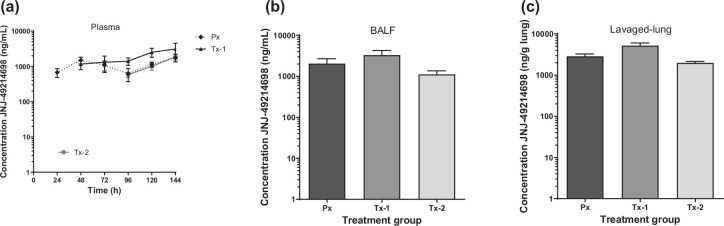
Exposure of JNJ-49214698 in different body compartments of neonatal lambs. (**a**) shows the daily plasma concentration (ng/ml) 24 h after the first dose and daily just before the next dose (C_trough_) to the end of the study. The compound reached a steady-state concentration in the plasma within 24 h of treatment and maintained that level throughout the study. (**b** and **c**) show JNJ-49214698 exposure in BALF (**b**) and lavaged lung (**c**) at necropsy (day 6 p.i.). Px *n*=4, Tx-1 *n*=5, Tx-2 *n*=5, RSV *n*=4, NoRSV *n*=3.

During a multicyclic RSV infection, small-molecule fusion inhibitors are thought to inhibit each new infection event at the time of viral fusion. The concentration of JNJ-49214698 was measured in BALF and homogenized lavaged-lung tissue. To assess the potential of JNJ-49214698 to distribute from the blood to the lungs of the neonatal lambs, we also measured its level in plasma. JNJ-49214698 was absorbed very quickly into the circulation and tended to accumulate slightly over time during the study period. The average steady-state concentration of JNJ-49214698 in plasma was in the range of 1770±435 and 3112±1441 ng/ml across the different treatment groups and was reached within 24–48 h after the first dose (Fig. 1a). About 24 h after the final dose, the level of JNJ-49214698 in the BALF of the group receiving prophylactic treatment (Px) or groups in which compound administration was started 1 (Tx-1) or 3 (Tx-2) days after viral inoculation was 2044±648, 3289±977 and 1109±252 ng/ml, respectively (Fig. 1b). The average lavaged-lung concentrations measured were 2867±403, 5211±841 and 1976±175 in Px Tx-1, and Tx-2 treatment groups, respectively, resulting in an approximate lung/plasma ratio of 1.4 to 1.8 and a good distribution of JNJ-49214698 in the BALF (Fig. 1c). Together, these results indicate a good distribution of JNJ-49214698 to different lung compartments. The protein binding of JNJ-49214698 in human plasma is moderate (fu=37%) with a calculated protein adjusted EC_90_ (paEC_90_)=4.6 ng/ml; the exposure levels in BALF and lung were well above the expected active level.

### Treatment with JNJ-49214698 reduces the incidence and duration of RSV-associated symptoms

**Fig. 2.**
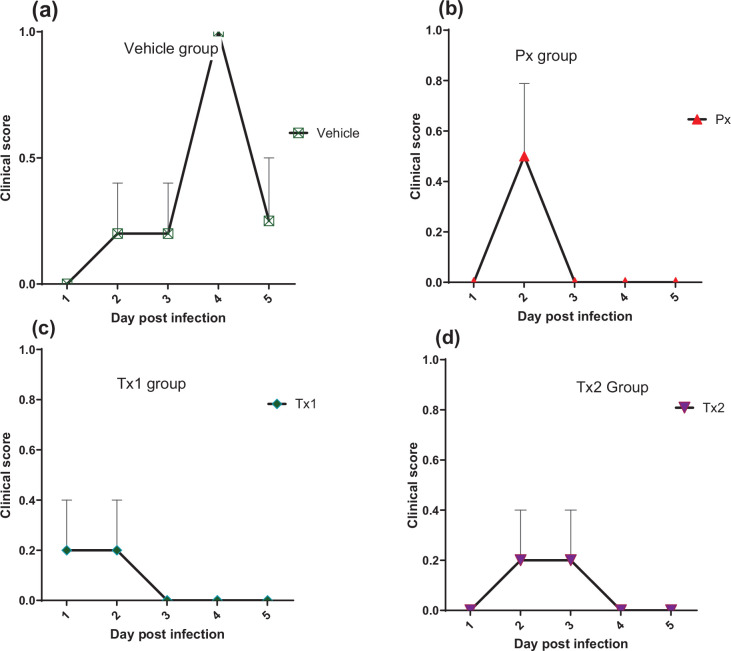
Clinical signs score in different groups. Data are shown as the average number of RSV-associated clinical signs observed in each group (wheezing, increased respiratory rate and expiratory effort). Animals were infected with RSV and received either vehicle only (Vehicle) *n*=4 (**a**), prophylaxis (Px) *n*=4 (**b**) or therapeutic treatment with JNJ-49214698, starting 1 (Tx-1) *n*=5 (**c**) or 3 (Tx-2) *n*=5 (**d**) days p.i. See the online supplement for a detailed table of the clinical signs observed.

Clinical signs of RSV infection in lambs, as in human infants, can be variable and sometimes difficult to assess. Despite this, RSV-associated respiratory distress can be observed in the animals as early as 1 day p.i., developing further in some animals into clear external signs of RSV-associated illness such as nasal discharge, wheezing or lethargy. By Day 1 p.i., most of the lambs in this study, except for the animals in the non-infected group, had already become lethargic as characterized by decreased activity (lower frequency of getting up and movement in general). The behaviour and respiratory-related symptoms in lambs of the vehicle-treated group (RSV and no treatment, *n*=4) steadily worsened during the following days (Fig. 2a). One lamb started to produce nasal discharge and displayed episodes of wheezing from Day 3 p.i. onwards, while its respiratory rate was clearly increased by Day 6. One lamb on Day 3 p.i. died and was found during post-mortem analysis with severe gross lesions consistent with RSV infection in its lungs. The other lambs survived until Day 6 when they were euthanized, but by Day 4, all four remaining animals from this group were lethargic.

In contrast, while by Day 3 p.i. three lambs in the Px group (*n*=4) showed fever, the remaining lambs in Px and Tx-1 groups were free of symptoms by Day 3 p.i. (Fig. 2b and c). Although less prominent, the incidence and duration of RSV-associated symptoms were also reduced in the lambs of the Tx-2 group (*n*=5) as compared to the vehicle-treated RSV-infected lambs (Fig. 2d).

In summary, these findings suggest that treatment with JNJ-49214698 has the potential to reduce the incidence and duration of the clinical manifestation of RSV disease in lambs. A summary of the clinical signs observed in lambs is highlighted in Table S1 (available in the online Supplementary Material).

### Treatment with JNJ-49214698 reduced RSV titre and infection in neonatal lambs

**Fig. 3.**
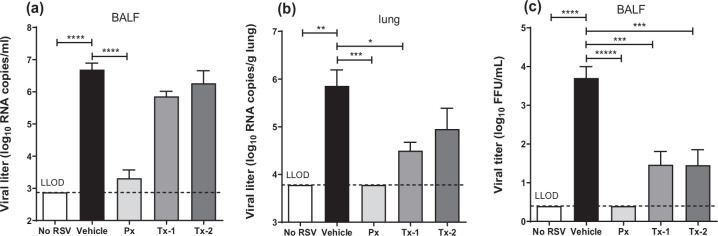
Effect of JNJ-49214698 on viral titres in BALF and lung tissue at Day 6 p.i. (**a**) the viral RNA titre as measured by qRT-PCR in BALF, (b) the viral RNA titre as measured by qRT-PCR in lung tissue, (**c**) number of infectious particles as measured by IFFU assay [26] with modification, all shown as average +SEM. Animals were infected with RSV and received either vehicle only (Vehicle), prophylaxis (Px) or therapeutic treatment with JNJ-49214698, starting 1 (Tx-1) or 3 (Tx-2) days p.i. Non-infected, vehicle-treated animals were indicated as No RSV. JNJ-49214698 reduces the viable viral particles in BALF and RSV RNA in both BALF and lung tissue. Statistical analysis was performed using one-way ANOVA and Dunnett’s post-hoc test for multiple comparison correction. *p-value<0.02; **p-value<0.002; ***p-value<0.001; ****p-value<0.0001. LLOD=lower limit of detection. Px *n*=4, Tx-1 *n*=5, Tx-2 *n*=5, RSV *n*=4, NoRSV *n*=3.

**Fig. 4.**
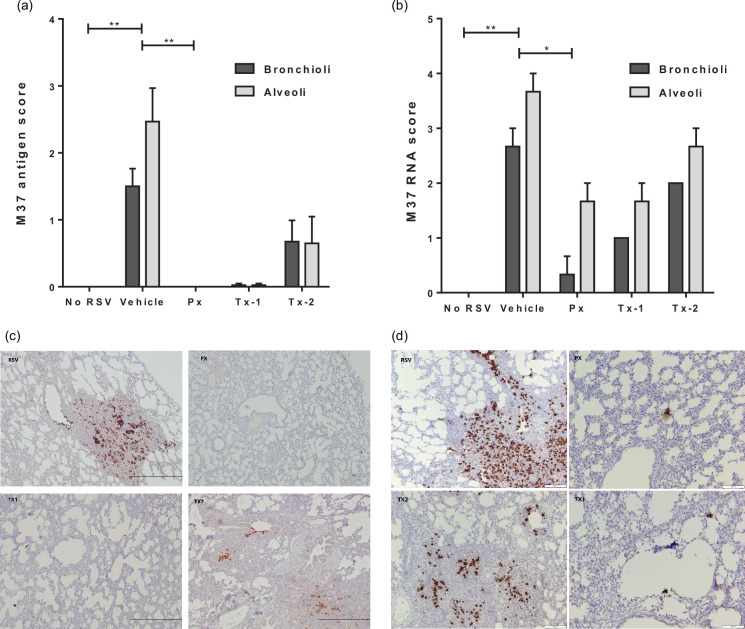
Effect of JNJ-49214698 on viral antigen and RNA in lung tissue at Day 6 p.i. (**a**) integer-based scoring of M37 antigen in lung tissue detected by immunohistochemistry, (**b**) integer-based scoring of M37 RNA in lung tissue detected by RNAscope. Scoring on *y*-axis represents the number of bronchioles or alveoli stained positive for M37 antigen (**a**) or RNA (**b**). Bars represent the average score per group +SEM. Animals were infected with RSV and received either vehicle only (Vehicle), prophylaxis (Px) or therapeutic treatment with JNJ-49214689, starting 1 (Tx-1) or 3 (Tx-2) days p.i. Non-infected, vehicle-treated animals were indicated as No RSV. (**c**) and (d) show representative images of M37 antigen (**c**) and M37 RNA (**d**) in the lung tissue of animals allocated to the different treatment groups. JNJ-49214698 reduced the RSV M37 antigen and RNA in the lungs of all JNJ-49214698-treated animals. Statistical analysis was performed by Kruskall–Wallis non-parametric test, followed by Dunn’s post-hoc test for multiple comparison correction. *P*-value bars are representative for both bronchioles and alveoli. *p-value<0.05; **p-value<0.01. Px *n*=4, Tx-1 *n*=5, Tx-2 *n*=5, RSV *n*=4, NoRSV *n*=3.

The presence of nucleoprotein RNA in BALF and lung samples was quantified by qRT-PCR. In BALF, the Px group showed a significant 3.4 log_10_ (*P*<0.0001) reduction of the viral RNA level in comparison to the RSV-infected vehicle group (Fig. 3a). In contrast, the viral RNA in the Tx-1 and Tx-2 groups was reduced less by approximately sevenfold and threefold compared to the vehicle-only treated animals. We could not detect viral RNA in the lung tissue of prophylactically treated animals and reductions in RSV RNA of approximately 23-fold in Tx-1 group (*P*<0.02) and 10-fold in Tx-2 compared to the vehicle group (Fig. 3b). The infectious viral titre in BALF was done and partially reported in a separate manuscript [25] in which the infectious viral titres were significantly reduced in all treated groups with no infective virus detected in the Px group that received prophylaxis with JNJ-49214698, similar to what we found in RSV mRNA in lung tissue. There were 34- and a 93-fold reduction in the infectious titre as compared to the RSV-infected control group, which was observed in the Tx-1 and Tx-2 groups, respectively (*P*<0.001) (Fig. 3c). To confirm the quantified viral titres in BALF and lavaged-lung tissue, we determined RSV (M37 fusion protein) antigen and RNA by immunohistochemical and RNAscope analysis, respectively. RSV antigen was present in infected airway epithelial cells of bronchi, bronchioles and alveoli and occasionally in alveolar macrophages. However, staining of RSV antigen was markedly reduced in all JNJ-49214698-treated groups compared to the RSV-infected control group (Fig. 4a and c). RSV antigen was highly decreased in the Tx-2 group as compared to the vehicle-treated group, and again, almost no RSV antigen was detected in both Px (*P*<0.01) and Tx-1 (*P*<0.05) treatment groups (Fig. 4a and c). When RSV RNA (M37 nucleoprotein) expression in lungs was determined by RNAscope, concomitant with the RNA levels as determined by qRT-PCR, there was a marked reduction of RSV RNA expression in all the compound-treated groups in both bronchioles and alveoli (Fig. 4b and d). Heavy brown staining of RSV RNA in RSV-infected non-treated lung sections was observed, and it was concentrated in the bronchioles and alveoli in the consolidated part of the lung (Fig. 4d). In contrast, only minimal staining was seen in the Px (*P*<0.05) and Tx-1 groups (Fig. 4d), aligning with immunohistochemical and qRT-PCR data.

### Treatment with JNJ-49214698 inhibits RSV-induced lung pathology and cellular immune response of neonatal lambs

**Fig. 5.**
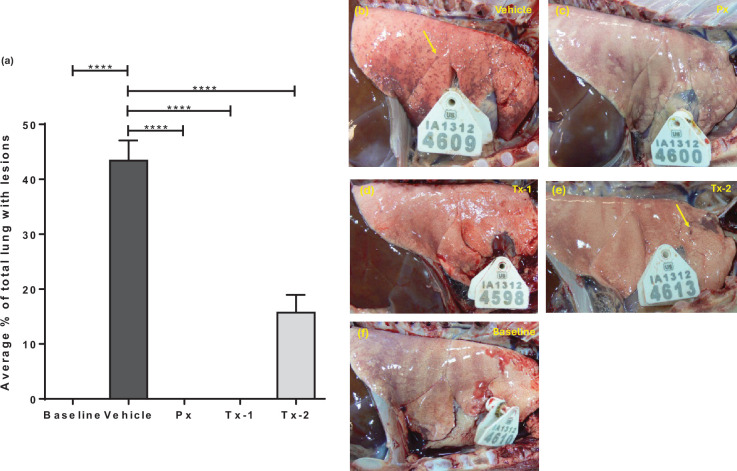
Effect of JNJ-49214698 on the development of gross lung lesions at Day 6 p.i. (**a**) shows the average +SEM% of lung surface affected by RSV-induced gross lesions in the different treatment groups. Animals were infected with RSV M37 and received either vehicle only (Vehicle), prophylaxis (Px) or therapeutic treatment with JNJ-49214689, starting 1 (Tx-1) or 3 (Tx-2) days p.i. Non-infected, vehicle-treated animals were indicated as No RSV. JNJ-49214698 significantly reduces the RSV gross lesions in the treated groups in comparison to the vehicle group. Statistical analysis was performed by one-way ANOVA and Dunnett’s post-hoc test for multiple comparison correction. ****p-value<0.0001. (**b**) shows representative images of lungs from an animal infected with RSV receiving vehicle only, prophylaxis with JNJ-49214698 or therapeutic treatment, starting 1 (Tx-1) or 3 (Tx-2) days p.i. and non-infected, vehicle-treated animals were indicated as baseline. Yellow arrows indicate variably sized dark red consolidated areas on the lung surface. Px *n*=4, Tx-1 *n*=5, Tx-2 *n*=5, RSV *n*=4, NoRSV *n*=3.

Approximately 43% of the lung surface of infected vehicle-treated animals had RSV-associated macroscopic lesions, which were multifocal dark red pinpoint foci of consolidation scattered randomly over the lung surface and deeply throughout the lung tissue (Fig. 5a and b). Moreover, gross lung lesions were evenly distributed over the left and right lobes. Prophylaxis (Px) or treatment starting 24 h after infection (Tx-1) completely abolished the formation of RSV-associated gross lung lesion (Fig. 5a and b). When treatment was delayed even as late as 3 days after infection (Tx-2), a significant reduction in the RSV-associated gross lesions was observed, dropping from 43% to 16% (*P*<0.0001) (Fig. 5b). As a negative control, no lesions were observed in the lungs of non-infected, vehicle-treated animals (Fig. 5b).

**Fig. 6.**
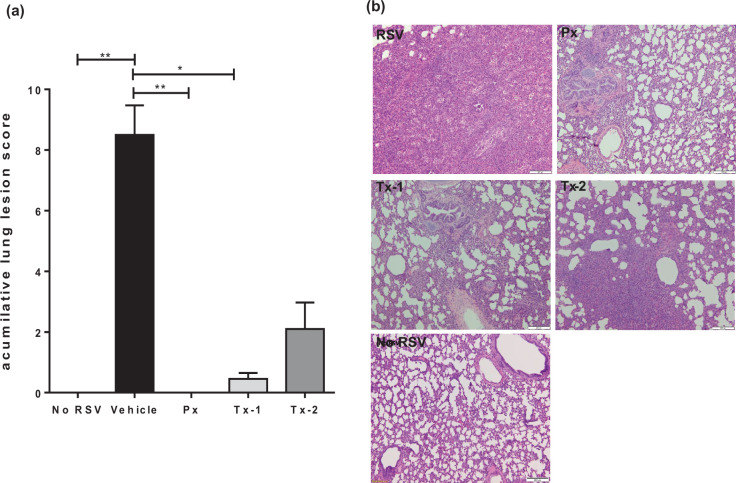
Effect of JNJ-49214698 on RSV-induced accumulated lung histopathology at Day 6 p.i. (**a**) shows the average +SEM of the accumulative RSV-induced microscopic lesions in the different treatment groups. Animals were infected with RSV M37 and received either vehicle only (Vehicle), prophylaxis (Px) or therapeutic treatment with JNJ-49214689, starting 1 (Tx-1) or 3 (Tx-2) days p.i. Non-infected, vehicle only-treated animals were indicated as No RSV. JNJ-49214698 reduces the pathological changes associated with RSV infection. Statistical analysis was performed by Kruskall–Wallis non-parametric test, followed by Dunn’s post-hoc test for multiple comparison correction. *p-value<0.05; **p-value<0.01. (**b**) shows representative images of the lung tissue sections from animals allocated to the different treatment groups. Px *n*=4, Tx-1 *n*=5, Tx-2 *n*=5, RSV *n*=4, NoRSV *n*=3.

To investigate the effect of JNJ-49214698 on lung tissue microscopically, we assessed the impact of the compound on the development of bronchiolitis, syncytial cell formation, epithelial necrosis and hyperplasia and inflammatory cell infiltration in the lungs. An accumulative histological lesion score was assigned by combining all the assessed parameters. Significant reductions of the accumulative score were seen across prophylactically treated animals or animals that received early treatment starting day 1 p.i. with JNJ-49214698 (*P*<0.01 and *P*<0.05, respectively) (Fig. 6a). Prophylaxis with JNJ-49214698 completely prevented the formation of microscopic lung lesions after RSV infection (Fig. 6a and b). Although the effect on the accumulative histologic score was least prominent in animals that received treatment with JNJ-49214698 as late as 3 days after infection, a prominent reduction as compared to the vehicle-treated group was still observed, with some animals displaying almost no microscopic lesions at all.

Typical RSV lesions that were present in the RSV-infected control group were multifocal interstitial pneumonia and bronchiolitis. The inter-alveolar wall thickened due to type II pneumocyte hyperplasia and lymphocyte infiltration (Fig. 6b). There was neutrophil infiltration in the bronchial and bronchiolar lumens and the alveolar lumen with multifocal and segmental areas of necrosis and sloughing of the epithelial cells lining bronchioles. Some bronchioles had modest thickening of epithelium due to hyperplasia. In addition, occasional multinucleated syncytial cells were present throughout the lung sections. In the lesions formed in the latter Tx-1 group, the formation of syncytial cells, epithelial necrosis or hyperplasia and infiltrating neutrophils were absent, while the other parameters assessed were markedly reduced, resulting in a prominent decrease of the accumulative histological lesion scores.

## Discussion

Currently, no effective direct antivirals are available for treating RSV-associated ALRTI. Even though a number of RSV inhibitors have reached early-stage clinical evaluation, no data are available yet demonstrating their clinical benefit in naturally infected patients suffering from RSV-associated ALRTI, and so uncertainty remains about the therapeutic treatment window and the impact of such molecules on severe RSV disease. Attempting to minimize the risk for intended late-stage clinical development, we, therefore, evaluated an experimental small-molecule RSV fusion inhibitor, JNJ-49214698, in neonatal lambs, a fully replicative animal model of RSV infection closely mimicking infant RSV disease [20]. This is a supplementary study to the previously published data [26].

The three treatment regimens chosen in the study all reflect realistic potential clinical drug administration regimens. The first group (Px) began to receive JNJ-49214698 1 day before viral nebulization to test the pre-exposure prophylactic effect of the compound, while the Day 1 p.i. treatment group (Tx-1) was used to test the effect of very early post-exposure treatment of an established infection. Previous RSV viral kinetic studies in our laboratory demonstrated that RSV replication in neonatal lambs is highest between viral inoculation and Day 3 p.i., reaching peak viral titre at Day 3, while RSV-associated lung pathology peaks at Day 6 p.i. [36]. Therefore, the Day 3 post-viral challenge treatment group (Tx-2) was used to test the effect of JNJ-49214698 on RSV-associated ALRTI when treatment is started close to peak viral titre, essentially the time patients seek first-line medical assistance and, on average, 1 day before they present to hospital [40,44].

The overall assessments demonstrate a strong pre-exposure protective effect of JNJ-49214698 against RSV infection. The prophylaxis (Px) group showed no gross/microscopic lung lesions and no viral antigen by immunohistochemistry (IHC). In addition, viral RNA levels significantly reduced close to undetectable levels and were mirrored by no detectable IFFU [26]. Only very occasional infection of individual lung cells, as demonstrated by the presence of viral RNA (by RNAscope) in these cells, was observed, consistent with earlier studies that showed that fusion inhibitors are effective in inhibiting syncytia formation by preventing transmission of the virus via cell-cell spreading [42,43, 45]. Our data are also consistent with the prophylactic efficacy of Synagis^®^, a humanized monoclonal antibody inhibiting the fusion protein of RSV [9]. In contrast to Synagis^®^, which lacks therapeutic efficacy, i.e. administration after the development of RSV infection [9], early (Tx-1) and late (Tx-2) therapeutic administration regimens with JNJ-49214698 displayed unambiguous evidence of efficacy in the neonatal lambs. Administration of JNJ-49214698 after the establishment of a multicyclic ALRTI strongly reduced the severity of the infection, as evidenced by the strong and statistically significant reduction of gross and microscopic changes in the lung and decreased infectious titre in BALF as compared to vehicle-only animals [26]. Consistent with these results, a considerable reduction of the viral RNA expression in lung tissue was measured. However, the significant reduction of infectious virus in BALF of therapeutically treated animals was not mirrored by a reduction of the viral RNA to the same extent. Although early administration of JNJ-49214698 starting 1 day after RSV infection resulted in better reduction and clearing of RSV infection as compared to the group that received late treatment starting at peak viral load, the efficacy results obtained in the latter group were clearly noticeable.

Together, the results of this study demonstrate that pre-exposure prophylaxis and treatment of an established RSV infection with a small-molecule RSV fusion inhibitor results in a significant reduction of viral replication as well as improvement of RSV-associated lung pathology. Moreover, our data suggest a favourable window within which to treat RSV infections in a community or hospital setting and contribute to the de-risking of late-stage clinical compound development pathways.

## supplementary material

10.1099/jgv.0.002056 Uncited Table S1.

## Abbreviations

ALRTI: acute lower respiratory tract infection
BALF: broncho alveolar lavage fluid
IFFU: infectious focus forming unit
IHC: immunohistochemistry
qRT-PCR: quantitative reverse transcription polymerase chain reaction
RSV: respiratory syncytial virus
