# Supplementary material for: Therapeutic efficacy of JNJ-49214698, an RSV fusion inhibitor, in RSV-infected neonatal lambs
Source: J Gen Virol. 2024 Dec 11;105(12):002056. doi: 10.1099/jgv.0.002056 (PMC11634040; doi:10.1099/jgv.0.002056)
Supplement: Uncited Table S1. [file jgv-105-02056-s001.pdf]

# Supplementary Data

## Materials and methods

**Compound and dosing**:

JNJ-49214698 was discovered and synthesized by Janssen Infectious Diseases (Beerse, Belgium). The compound was formulated in 10% acidified hydroxypropyl-β-cyclodextrin [10% HP-β-CD + HCl, pH 2 (vehicle)] at 6.25 mg/mL prior to dosing and stored throughout the study at 4°C. The compound was dosed orally by catheter-mediated orogastric gavage at 4 mL/kg body weight (25 mg/kg) once daily. Dose selection in this study was aimed to reach the highest possible, safe exposure in the animals to maximize the likelihood of obtaining efficacy while avoiding toxic side effects. A daily oral dose of 25 mg/kg was selected based on the antiviral activity of JNJ-49214698 [$EC_{50}$ = 0.4 ng/mL (0.8 nM) and $EC_{90}$ = 2.4 ng/mL (4.8 nM)], JNJ-49214698 exposure levels obtained in neonatal lambs at different doses during a separate pharmacokinetic (PK) study and an observed lack of toxicity of JNJ-49214698 at least until $C_{max}$ = 17,167 ng/mL and $AUC_{0\text{-}24h}$ = 139,993 ng.h/mL in a 5-day repeated dose rat tolerance study.

**Animals**:

Twenty-one colostrum-deprived neonatal lambs (Suffolk, Polypay, Dorsett cross) aged 1-3 days and 2-7 kg body weights were obtained for this experiment. Animal use was approved by the Institutional Animal Care and Use Committee of Iowa State University. RSV-infected lambs were kept in a separate room from the non-infected animals in the Livestock Infectious Disease Isolation Facility (LIDIF). These rooms have separate ventilation units as well as separate entrances and exits to avoid any cross-contamination between infected and non-

infected lambs. Lambs were fed an iodide-free lamb milk replacer diet (Milk Products Inc., Chilton, WI, USA) [1] and were treated with Naxcel (Ceftiofur sodium, Pfizer) intramuscular once daily to reduce/prevent secondary bacterial infections.

**Experimental design**:

Lambs were randomly assigned to five different groups. Three groups (Px, Tx-1, and Tx-2) were infected with RSV and treated with JNJ-49214698. The first group (Px, n = 4) was treated prophylactically 1 day before RSV challenge and then daily afterward up until Day 5 post infection (p.i.). The second (Tx-1, n = 5) and the third (Tx-2, n = 5) groups were treated one day and three days after viral challenge and daily afterward up until Day 5 p.i., respectively. The vehicle group (n = 4), serving as a positive RSV control group, was infected with RSV but received treatment with vehicle only. The No RSV group (n = 3), served as negative RSV control group, was aerosolized with RSV-free, HEp-2 cell-conditioned media, and received vehicle. All lambs were euthanized at Day 6 p.i. and all endpoints were measured after euthanasia.

**RSV infection**:

Lambs were infected with RSV strain M37, purchased from Meridian BioSciences (Memphis, TN, USA). This strain is a wild type A RSV isolated from the respiratory secretions of an infant hospitalized for bronchiolitis [2, 3]. In our laboratory, M37 was grown in HEp-2 cells and stored at -80°C in media containing 20% sucrose [4]. PARI LC Sprint™ nebulizers were used to administer virus or cell-conditioned control media (lacking RSV) to each lamb [5]. Six mL of $1.27 \times 10^7$ Infectious Forming Unit (IFFU)/mL in media containing 20% sucrose or cell-conditioned mock media (also containing 20% sucrose) was nebulized

to each lamb over the course of 25-30 minutes resulting in the total inhalation of about 3 mL by each lamb.

**Animal monitoring for the appearance of clinical signs and the clinical score:**

Animals were monitored for clinical signs at the beginning of the study right before viral inoculation (Day 0: all animals scored 0) and immediately after each administration of vehicle or test article throughout the course of the study. In addition to monitoring their behavior, respiratory associated clinical signs (respiratory rate, wheezing, expiratory effort) were measured. Because clinical signs are variable in lambs and challenging to score in terms of severity, an accumulative clinical score to summarize the overall distress per group was applied by adding up the number of the scored clinical signs observed in each of the individual lambs and then averaging the daily obtained sums for the respective groups to normalize for group size. [6, 7]

**Blood sampling for PK analysis**:

Blood samples (1.5 - 2 mL) were collected from the jugular vein pre-dose (just before the first dose) and at 24 h following each dose until 144 h for PK analyses. Blood was dispensed into 3 mL blood collection tubes containing $K_2EDTA$ anticoagulant. Blood samples were kept at room temperature prior to centrifugation. The blood samples were then centrifuged at 1,600 x g for 10 min at 4°C to obtain the plasma. Plasma was stored in 2 mL cryovials at -80°C.

**Lung collection and processing**:

The thorax was opened, lungs removed, and gross lesions were scored as performed previously (40). The lungs were also photographed *in situ* and *ex vivo*. After removal, each lung lobe's percentage of parenchymal involvement was scored before the bronchoalveolar lavage fluid (BALF) collection procedure. Left and right lungs were then separated, and each lobe excised. Tissue samples were collected from each lung lobe of all animals. In brief, one sample from each lobe not destined for BALF collection (i.e. 4 lobes - Right Cranial, Left Cranial, Left Middle and Left Caudal) were snap-frozen in liquid nitrogen for qRT-PCR. Two samples from each of these lobes were placed in tissue cassettes and put in 10% neutral-buffered formalin (NBF) for histological and immunohistochemical analysis. Representative lung samples from each of these lobes were also placed into a cryovial and immediately snap-frozen in liquid nitrogen, then transferred to -80°C for storage and shipped on dry ice pellets for PK analysis of JNJ-49214698 compound at Janssen.

**BALF collection**:

BALF samples from each animal were collected immediately after euthanasia on Day 6 p.i. from the right middle and right caudal lobes as performed previously in our laboratory [6]. Briefly, the excised lung lobes were instilled with 5 mL of cold DMIM (42.5% Iscove's modified Dulbecco's medium, 7.5% glycerol, 1% heat-inactivated FBS, 49% DMEM, and 5 μg/ml kanamycin sulfate). 100 μL of the right caudal lobe BALF was added to 1 mL TRIzol (Invitrogen) and kept at – 80 °C for the qRT-PCR assay to assess RSV mRNA, and the rest of BALF sample was placed on ice and used within 2 h for infectious focus-forming unit (IFFU) assay to assess the infectious RSV titer. The right middle lobe BALF sample was

placed at -80°C for storage and shipped on dry ice pellets to determine JNJ-49214698 and blood urea nitrogen concentrations at Janssen.

**Quantification of JNJ-49214698 exposure**

Samples of plasma (50 µL), lung homogenate (50 µL) and BALF (50 µL) were analyzed for JNJ-49214698 using a method based on protein precipitation and HPLC/MS/MS analysis. DMSO (50 µL) and acetonitrile (500 µL) were added to each sample. Samples were mixed thoroughly (mechanical shaking for 10 min) and then centrifuged (at 5000 × *g* for 10 min at 15°C). An aliquot (400 µL) of the resulting supernatant was transferred to a 96-well plate and assayed for JNJ-49214698 concentrations using HPLC/MS/MS employing positive-ion electrospray ionization (Sciex API 4000) and a Waters ACQUITY UPLC C18 1.7um (50 x 2.1 mm i.d.) column. Elution was achieved at a flow rate of 0.8 mL/min with a gradient of 0.1% FA and acetonitrile. The lower limit of quantification was 1 ng/mL for plasma, 50 ng/g for lung, and 1 ng/mL for BALF. The assay was linear up to 20,000 ng/mL for plasma, 5,000 ng/mL for BALF and 100,000 ng/g for lung. Samples of plasma and BALF were also analyzed for concentrations of urea in order to calculate the dilution of BALF on sample collection. To a separate sample of plasma and BALF (50 µL), 50 µL $H_2O$/acetonitrile (50/50), 50 µL internal standard, 200 µl acetonitrile was added. Samples were mixed thoroughly (mechanical shaking for 10 min), and then centrifuged (5000 × *g* for 10 min at 15°C). An aliquot (200 µL) of the supernatant was transferred to a 96-well plate and evaporated at 40°C with nitrogen. An aliquot (200 µL) of camphanic chloride (1 mg/mL in acetonitrile) was added for derivatization. Samples were mixed thoroughly (mechanical shaking for 10 min) and incubated for 90 min at 37C. After incubation, 100 µL $H_2O$ was added and assayed for urea concentrations using HPLC/MS/MS employing positive-ion electrospray ionization

(Sciex API 4000) and a Waters ACQUITY UPLC HSS T3 (50 x 2.1 mm i.d.) column. Elution was achieved at a 1 mL/min flow rate with a gradient of 0.01M Ammonium formate (pH = 3) and acetonitrile. The lower limit of quantification was 2 μg/mL for plasma and BALF. The assay was linear up to 10,000 μg/mL.

**Quantitative reverse transcription polymerase chain reaction (qRT-PCR):**

Tissue samples from right and left cranial, left middle and left caudal lung lobes (0.3–0.4 g of each lobe) were homogenized in TRIzol (Invitrogen, Carlsbad , CA) for total RNA isolation according to manufacturer's instructions and as previously described [4, 6, 8]. Briefly, RNA isolation was followed by DNase treatment (Ambion, TURBO DNase, Austin, TX) at 1:10 dilution in combination with RNaseOUT (Invitrogen) and nuclease-free water (GIBCO/Life Technologies, Carlsbad, CA). Spectrometry (Beckman Scientific, Indianapolis, IN) was used to assess each RNA sample isolate at a dilution of 1:50 to measure sample purity and quantity (A260nm/A280nm all >1.95). Agilent Bioanalyzer 2100 analyses of the RNA isolates gave RNA Integrity Number values > 8.0. qRT-PCR was carried out using One-Step Fast qRT-PCR Kit master mix (Quanta, BioScience, Gaithersburg, MD) in a StepOnePlus™ qPCR machine (Applied Biosystems, Carlsbad, CA) in conjunction with PREXCEL-Q assay-optimizing calculations [9, 10]. Primers and probes for RSV M37 nucleoprotein were designed based on RSV accession number M74568. Forward primer: 5′-GCTCTTAGCAAAGTCAAGTTGAACGA; reverse primer: 5′-TGCTCCGTTGGATGGTGTATT; hydrolysis probe: 5′-6FAM-ACACTCAACAAAGATCAACTTCTGTCATCCAGC-TAMRA. Each 1:10-diluted total RNA sample was further diluted so that each final qRT-PCR contained 0.784 ng total RNA/μL; as determined to be optimal by PREXCEL-Q [11]. Thermocycling conditions were

5 minutes at 50°C, 30 seconds at 95°C, and 45 cycles of 3 seconds at 95°C and 30 seconds at 60°C. Samples and standards were assessed in duplicate and each qRT-PCR quantification cycle (Cq) was converted to a relative initial quantity ($X_o$) based on a standard curve using the following equation: $X_o = E_{AMP}^{(b-Cq)}$, where $E_{AMP}$ and b are the PCR exponential amplification efficiency value and the y-intercept, respectively, from a sample mixture derived standard curve for RSV M37 nucleoprotein mRNA. The efficiency-corrected delta Cq ($E_{AMP}^{\Delta Cq}$) method was used for qRT-PCR quantification calculations. Results were normalized to total tissue RNA loaded per reaction (identical for all reactions). No-RT control reactions proved negative for RSV M37. qRT-PCR was demonstrably free of inhibition based on preliminary dilution threshold analyses as per the PREXCEL-Q method for qPCR [11, 12].

**Hematoxylin-eosin staining and histological scoring of lung sections**:

Hematoxylin-eosin stained sections were examined via light microscope as described previously [6] with some modification. Lung lesions were scored according to an integer-based score of 0-4 for each parameter (bronchiolitis, syncytial cells, epithelial necrosis, epithelial hyperplasia, peribronchial lymphocytic infiltration, perivascular lymphocytic infiltration, neutrophils), with 4 as the highest score. Then a final score (accumulative histo lesion score) was assigned by adding up the scores from the seven individual parameters, resulting in final accumulative scores ranging from 0-28, representing the total RSV-associated lesion in each tissue section. The scale used to assess the pathological changes briefly for bronchiolitis scale: 0 = no remarkable lesions, 1 = minimal detectable lesion (epithelial degeneration in one or a few bronchioles per 20 X field), 2 = epithelial degeneration involving less than 10% of the airway lumen; minimal neutrophils and cell

debris; adventitial lymphocytes in multiple bronchioles, 3 = epithelial degeneration involving more than 10-50% of the airway lumen with cell debris and neutrophils; adventitial lymphocytes; multiple bronchioles, 4 = circumferential bronchiolitis with dense adventitial lymphocytes; multiple bronchioles. For syncytial cells scale: 0 = none, 1 = one distinct syncytial cell, 2 = up to three in three 20x fields, 3 = more than three in three fields, 4 = numerous. Epithelial necrosis and epithelial hyperplasia scale: 0 = none, 1 = minimally detectable in one or a few bronchioles per 20x per field, 2 = 10% of the bronchioles in multiple airways per field, 3 = 10-50% of the bronchioles in multiple airways per field, 4 = circumferential in multiple airways. Neutrophils scale (in bronchi, bronchioles or alveoli): 0 = none, 1 = minimally detectable, 2 = 10 or less neutrophils in one or a few airways/alveoli, 3 = 10 or more neutrophils in several airways/alveoli, 4 = 10 or more involving many or most airways/alveoli. The same scale was used for peribronchiolar lymphocytic infiltrates and perivascular lymphocytic infiltrates: 0 = none, 1 = earliest detectable lymphocytic infiltration in the adventitia, 2 = segmental to circumferential infiltration, 3 = circumferential infiltrates that expand more than three cells wide, 4 = circumferential infiltrates that form nodules.

**Immunohistochemistry (IHC) of lung sections**:

IHC was used to determine the distribution of RSV antigens, as described previously [6, 13, 14]. Briefly, after deparaffinization and rehydration of the formalin-fixed paraffin-embedded tissue sections, antigen retrieval was performed by placing the slides with tissue sections in pH 9.0 10mM TRIZMA base, 1mM EDTA buffer and 0.05% Tween 20 and boiling under pressure for up to 15 minutes. Polyclonal goat anti-RSV antibody (Millipore/Chemicon, Temecula, CA; Cat. No. AB1128) was used as the primary antibody after two blocking steps, the first with 3% bovine serum albumin in Tris-buffered saline + 0.05% Tween 20 (TBS-T)

and the second with 20% normal swine serum in TBS-T, 15 minutes each. The primary antibody was followed by a biotinylated rabbit anti-goat secondary antibody (KP&L; Cat. No. 16-13-06). Signal development was done by using 1:200 dilution of streptavidin-horseradish peroxidase (Invitrogen; Cat. No. 43-4323) for 30 minutes, followed by incubation with Nova Red chromagen solution (Vector; Cat. No. SK-4800). The positive signal was quantified in both bronchioles and alveoli for each tissue section, and a score of 0-4 was assigned according to an integer-based scale of 0 = no positive alveoli/bronchioles, 1 = 1-10 positive alveoli/bronchioles, 2 = 11-39 positive alveoli/bronchioles, 3 = 40-99 positive alveoli/bronchioles, 4 = >100 positive alveoli/bronchioles.

**RNAscope**:

Formalin-fixed paraffin-embedded (FFPE) IHC tissue sections were used for the RNAscope detection of RSV mRNA *in situ*. A probe was designed for the hRSV M37 nucleoprotein gene (Probe-V-RSV-NP, Advance Cell Diagnostic, Catalog number 439866). This probe was designed to target the 8-1111 base region of the nucleoprotein gene of accession number KM360090.1 CDS sequence (1114-2289). The assay was performed according to the manufacturer's manual (user manual document number 320497; RNAscope® 2.0 HD Detection Kit (BROWN) User Manual PART 2). Sections were examined under light microscope, and the number of bronchioles and alveoli containing the positive signal were counted. The number of positive bronchioles and alveoli per tissue section was then assigned a score according to the simple integer-based scale of 0 = no positive alveoli/bronchioles, 1 = 1-10 positive alveoli/bronchioles, 2 = 11-39 positive alveoli/bronchioles, 3 = 40-99 positive alveoli/bronchioles, 4 = >100 positive alveoli/bronchioles.

**Infectious focus-forming unit (IFFU) assay**:

Viral titers in BALF from the right caudal lobe were determined by IFFU assay [6]. BALF samples were spun down for 5 minutes in a centrifuge at 3,000 x g to pellet large debris. Approximately 800–850 µL of each supernatant was collected and then spun through 850 µL-capacity 0.45 µm Costar SPIN-X filter (microcentrifuge 15,600 x g) for 5 minutes. The resulting clear BALF samples were applied to HEp-2 cells grown to 70% confluence in 12-well culture plates (Fisher Scientific, Hanover Park, IL) at full strength and three serial dilutions (1:10, 1:100, and 1:1000), all tested in triplicate. The BALF samples in the wells were diluted with DMEM media (Mediatech, Inc., Manassas, VA) supplemented with 10% with heat-inactivated fetal bovine serum (FBS) (Atlanta Biologicals, Atlanta, GA) and 50 µg/mL kanamycin sulfate (Invitrogen/Life Technologies). Plates were incubated for 1 hr at 37°C, 5% $CO_2$, then 1 mL of media was added to each well and plates were returned to the incubator. After a 48 hr incubation, wells were fixed with cold 60% acetone/40% methanol solution for 1 minute. Wells were then rehydrated with TBS-T, and blocked with 3% BSA solution for 15 minutes, followed by overnight incubation with primary polyclonal goat anti-RSV (all antigens) antibody (EMD Millipore Corporation, Billerica, MA, USA). The next day, plates were washed with TBS-T then incubated for 1 hr with secondary antibody (Alexa Fluor® 488) F(ab')2 fragment of rabbit anti-goat IgG (H+L) (Molecular Probes/Life Technologies). Plates were rinsed and examined under inverted fluorescence microscopy using the FITX/GFP filter (Olympus CKX41, Center Valley, PA), and the total number of IFFUs (which is defined here as 3 or more fluorescing cells) were counted for each well. IFFU/mL calculations were obtained by multiplying the average number resulting from triplicate well counts by the initial BALF sample dilution factor and multiplying that value

by 5 to obtain counts/mL since 1,000 µL/200 µl (the actual sample applied for each well) equals 5.

**Statistical analysis:**

Statistical analysis was completed by using the Kruskall-Wallis test for non-parametric parameters such as accumulative microscopic lesion scoring, immunohistochemistry and RNAscope integer-based scores, followed by Dunn's post-hoc test for multiple comparisons. One-way ANOVA followed by Dunnett multiple comparisons test was used to compare the treated groups to the RSV-infected non-treated control group for gross lesion scores and viral titer analyses by qRT-PCR and IFFU assays.

**Table 1: clinical signs observed in lambs in different groups throughout the study period**

| RSV-associated symptoms | | | | | | |
|---|---|---|---|---|---|---|
| **Group** | **Animal ID** | **Day 1** | **Day 2** | **Day 3** | **Day 4** | **Day 5** |
| **Vehicle** | 1 | | | W | L | R |
| | 2 | | | | D | |
| | 3 | | ND | | L | |
| | 4 | | | | L | |
| | 5 | | | | L | |
| **Px** | 6 | | R | | | |
| | 7 | | R | | | |
| | 8 | | | | | |
| | 9 | | | | | |
| **Tx-1** | 11 | W | R | | | |
| | 12 | | | | | |
| | 13 | | | | | |
| | 14 | | | | | |
| | 15 | | | | | |
| **Tx-2** | 16 | | | | | |
| | 17 | | | | | |
| | 18 | | W | | | |
| | 19 | | | | | |
| | 20 | | | Ee | | |
| No | 21 | | | | | |
| | 22 | | | | | |
| RSV | 23 | | | | | |

W = wheeze, L = lethargy, R = increased respiratory rate, Ee = expiratory effort, D = Dead, ND = nasal discharge.

**References:**


1. **Derscheid RJ, van Geelen A, Berkebile AR, Gallup JM, Hostetter SJ, *et al.*** Increased Concentration of Iodide in Airway Secretions Is Associated with Reduced Respiratory Syncytial Virus Disease Severity. *Am J Respir Cell Mol Biol* 2013;50:389–397.

2. **DeVincenzo JP, Wilkinson T, Vaishnaw A, Cehelsky J, Meyers R, *et al.*** Viral Load Drives Disease in Humans Experimentally Infected with Respiratory Syncytial Virus. *Am J Respir Crit Care Med* 2010;182:1305–1314.

3. **Kim Y-I, DeVincenzo JP, Jones BG, Rudraraju R, Harrison L, *et al.*** Respiratory Syncytial Virus Human Experimental Infection Model: Provenance, Production, and Sequence of Low-Passaged Memphis-37 Challenge Virus. *PLOS ONE* 2014;9:e113100.

4. **Derscheid RJ, van Geelen A, McGill JL, Gallup JM, Cihlar T, *et al.*** Human Respiratory Syncytial Virus Memphis 37 Grown in HEp-2 Cells Causes more Severe Disease in Lambs than Virus Grown in Vero Cells. *Viruses* 2013;5:2881–2897.

5. **Grosz DD, van Geelen A, Gallup JM, Hostetter SJ, Derscheid RJ, *et al.*** Sucrose stabilization of Respiratory Syncytial Virus (RSV) during nebulization and experimental infection. *BMC Res Notes* 2014;7:158.

6. **Larios Mora A, Detalle L, Van Geelen A, Davis MS, Stohr T, *et al.*** Kinetics of Respiratory Syncytial Virus (RSV) Memphis Strain 37 (M37) Infection in the Respiratory Tract of Newborn Lambs as an RSV Infection Model for Human Infants. *PLoS ONE*;10. Epub ahead of print 7 December 2015. DOI: 10.1371/journal.pone.0143580.

7. Determinination of the viral kinetics of human RSV M37 strain in newborn lambs for use in therapeutic testing and characterization of clinical signs and sickness behavior. https://dr.lib.iastate.edu/entities/publication/ec1c34ab-8b17-4b04-99be-299cd54b85ac (accessed 10 September 2024).

8. **Derscheid RJ, Gallup JM, Knudson CJ, Varga SM, Grosz DD, *et al.*** Effects of Formalin-Inactivated Respiratory Syncytial Virus (FI-RSV) in the Perinatal Lamb Model of RSV. *PLOS ONE* 2013;8:e81472.

9. **Gallup JM, Ackermann MR**. The ‘PREXCEL-Q Method’ for qPCR. *Int J Biomed Sci IJBS* 2008;4:273–293.

10. **Sow FB, Gallup JM, Sacco RE, Ackermann MR**. Laser Capture Microdissection Revisited as a Tool for Transcriptomic Analysis: Application of an Excel-Based qPCR Preparation Software (PREXCEL-Q). *Int J Biomed Sci IJBS* 2009;5:105–124.

11. **Polack FP, Teng MN, L.Collins P, Prince GA, Exner M, *et al.*** A Role for Immune Complexes in Enhanced Respiratory Syncytial Virus Disease. *J Exp Med* 2002;196:859–865.

12. **Delgado MF, Coviello S, Monsalvo AC, Melendi GA, Hernandez JZ, *et al.*** Lack of antibody affinity maturation due to poor Toll-like receptor stimulation leads to enhanced respiratory syncytial virus disease. *Nat Med* 2009;15:34–41.

13. **Olivier A, Gallup J, De Macedo MMMA, Varga SM, Ackermann M**. Human respiratory syncytial virus A2 strain replicates and induces innate immune responses by respiratory epithelia of neonatal lambs. *Int J Exp Pathol* 2009;90:431–438.

14. **Meyerholz DK, Grubor B, Fach SJ, Sacco RE, Lehmkuhl HD, *et al.*** Reduced clearance of respiratory syncytial virus infection in a preterm lamb model. *Microbes Infect* 2004;6:1312–1319.
